# Topology Consistency of Disease-specific Differential Co-regulatory Networks

**DOI:** 10.1186/s12859-019-3107-8

**Published:** 2019-11-06

**Authors:** Maryam Nazarieh, Hema Sekhar Reddy Rajula, Volkhard Helms

**Affiliations:** 1 Center for Bioinformatics, University of Saarland, Saarbruecken, Germany; 2 Graduate School of Computer Science, University of Saarland, Saarbruecken, Germany

**Keywords:** Topology consistency, TF-miRNA co-regulatory networks, TFmiR, Minimum dominating set, Minimum connected dominating set

## Abstract

**Background:**

Sets of differentially expressed genes often contain driver genes that induce disease processes. However, various methods for identifying differentially expressed genes yield quite different results. Thus, we investigated whether this affects the identification of key players in regulatory networks derived by downstream analysis from lists of differentially expressed genes.

**Results:**

While the overlap between the sets of significant differentially expressed genes determined by DESeq, edgeR, voom and VST was only 26% in liver hepatocellular carcinoma and 28% in breast invasive carcinoma, the topologies of the regulatory networks constructed using the TFmiR webserver for the different sets of differentially expressed genes were found to be highly consistent with respect to hub-degree nodes, minimum dominating set and minimum connected dominating set.

**Conclusions:**

The findings suggest that key genes identified in regulatory networks derived by systematic analysis of differentially expressed genes may be a more robust basis for understanding diseases processes than simply inspecting the lists of differentially expressed genes.

## Background

RNA-Seq or whole transcriptome shotgun sequencing quantifies the abundance of RNA in a biological sample. Read counts refer to the number of reads mapping to gene segments in the DNA sequence.

Despite a high correlation between gene expression profiles using the same set of samples, RNA-Seq is capable of detecting low abundance transcripts and allows for the detection of more differentially expressed (DE) genes with higher fold-changes than microarray data [1].

A typical differential expression analysis of RNA-Seq data starts with normalizing raw counts and dispersion estimation. Then a statistical test is performed to determine which of the observed differences in read counts between two groups are statistically significant. The results returned by differential expression analysis typically in terms of *p*-values reject or accept a certain null hypothesis which signifies that the mean values of the two groups are equal or that the read counts follow the same distribution. To obtain accurate results, an assumption about the distribution of the underlying data is required. A t-test which is widely used to process microarray data assumes that the data has a normal distribution. This assumption does not hold for RNA-Seq data with discrete values. Several data distributions have been suggested to model RNA-Seq values. Among them, Poisson distribution and Negative Binomial (NB) distribution are used most often. The Poisson distribution does not account for over-dispersion in the data and assumes that mean and variance are equal which leads to high false discovery rates. Therefore, the NB distribution that considers both mean and dispersion parameters is typically preferred to model RNA-Seq data. Although, several methods such as DESeq [2] and edgeR [3] assume that RNA-Seq data can be modelled by the NB distribution, each of them uses a different approach to estimate the model parameters, mean and dispersion. This leads to different results for DE genes. The problem gets more severe when the methods make different assumptions about the underlying data distribution. Soneson and Delorenzi [4] conducted a comprehensive comparison between the results of eleven differential expression analysis methods which take RNA-Seq read counts as input on both simulated and real data. There appears to be no general consensus among the DE genes found by the different methods [4]. This may have clear implications on any downstream analysis.

In this work, we show that topological features are highly consistent despite the large number of exclusive DE genes identified by different methods. Here, we selected the four methods DESeq, edgeR, voom and VST from the above-mentioned methods which all take read counts as input and return *p*-values. We applied these methods to liver hepatocellular carcinoma (LIHC) and breast invasive carcinoma (BRCA) datasets including matched tumor and normal samples from The Cancer Genome Atlas [5, 6] and determined the significant DE genes. After illustrating the relatively small overlap among their results, we showed that key players are highly consistent among different methods even when differing sets of DE genes are provided as input. For this, we used the TFmiR webserver [7] to construct disease-specific TF-miRNA co-regulatory networks for the sets of identified DE genes. Then we identified two sets of genes that serve as key players of the DE genes in slightly different topological ways, namely a minimum dominating set (MDS) and a minimum connected dominating set (MCDS), see [8].

## Results

### Inference of dE genes

**Fig. 1:**
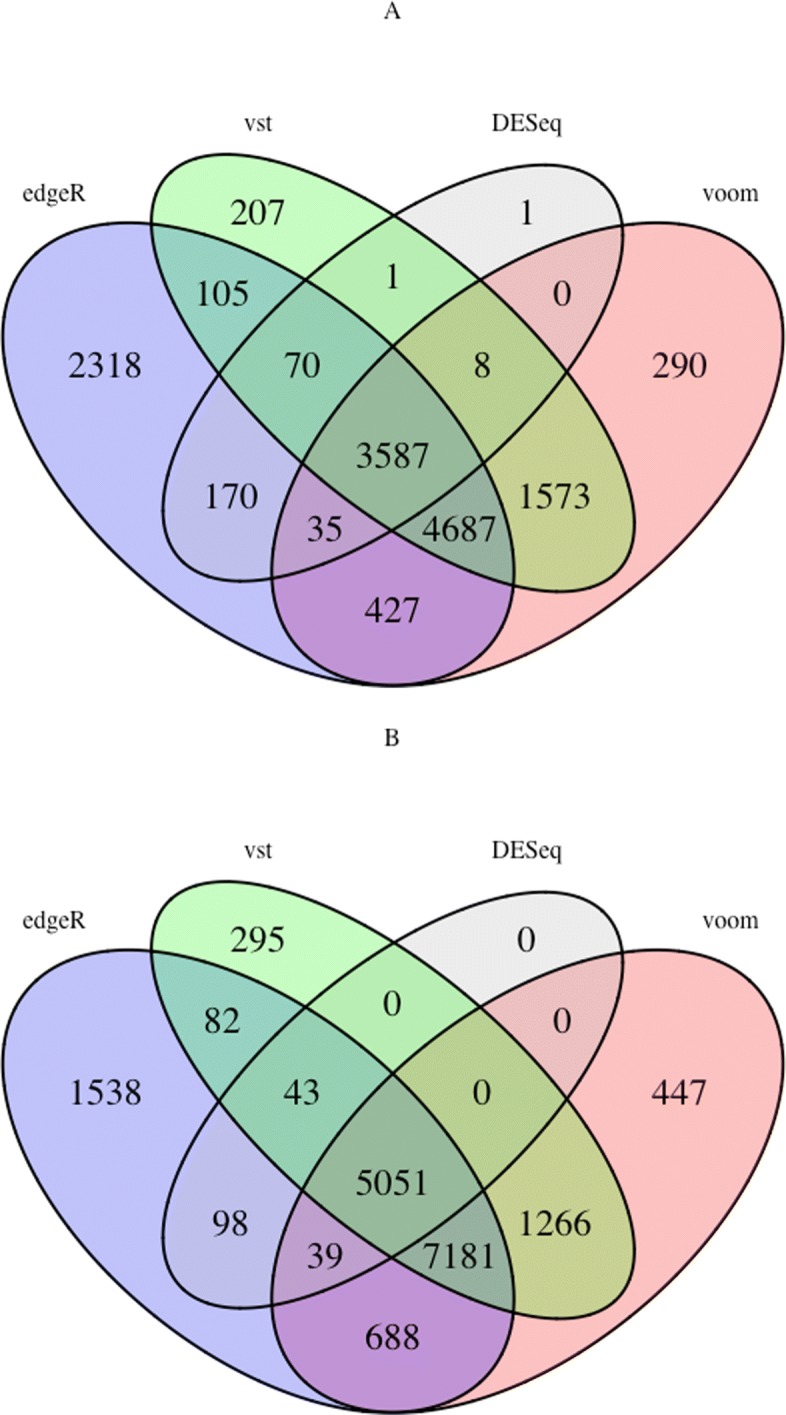
Overlap of the DE genes of DESeq with edgeR, voom and VST. **a** Venn diagram describing the number of overlapped DE genes between the results of DESeq with edgeR, voom and VST for the LIHC dataset. **b** same for the BRCA dataset. The Venn diagrams were visualized using the R package VennDiagram [9]

The processed matching tumor-normal samples for LIHC and BRCA consisted of 100 and 226 samples with 20501 genes, respectively. The data were given as input to the R packages DESeq, edgeR, voom and VST. Based on the adjusted *p*-value threshold of 0.05, we determined sets of DE genes. The number of significant DE genes for the LIHC dataset with DESeq, edgeR, voom and VST were 3872, 11399, 10610 and 10238, respectively and for the BRCA dataset 5231, 14722, 15559 and 13918, respectively. Venn diagrams in Fig. 1 show the number of genes which are common between these methods. The overlap between all methods is only 26% and 28%, respectively. This largely stems from the fact that DESeq identifies far fewer DE genes than the other 3 methods. Additional file 1:Table S1 lists the pairwise percentage overlap (percentage overlap or overlap coefficient between two sets *X* and *Y* is defined as overlap$(X,Y) = \frac {|X \cap Y |}{min(|X|,|Y|)}$) between the identified DE genes derived by the aforementioned methods with the number of exclusive ones among them for the LIHC dataset. The pairwise overlap coefficient between the results of two DE methods is quite high, ranging from 82% to 89% between edgeR and voom/VST results to 100% between edgeR and DESeq. Nonetheless, the results always differ by a considerable number of exclusive DE genes (1135 - 9489) that are only identified by one method but not the other one. Similar results were obtained for the BRCA dataset, see Additional file 1: Table S2.

### Reconstructed networks

**Fig. 2:**
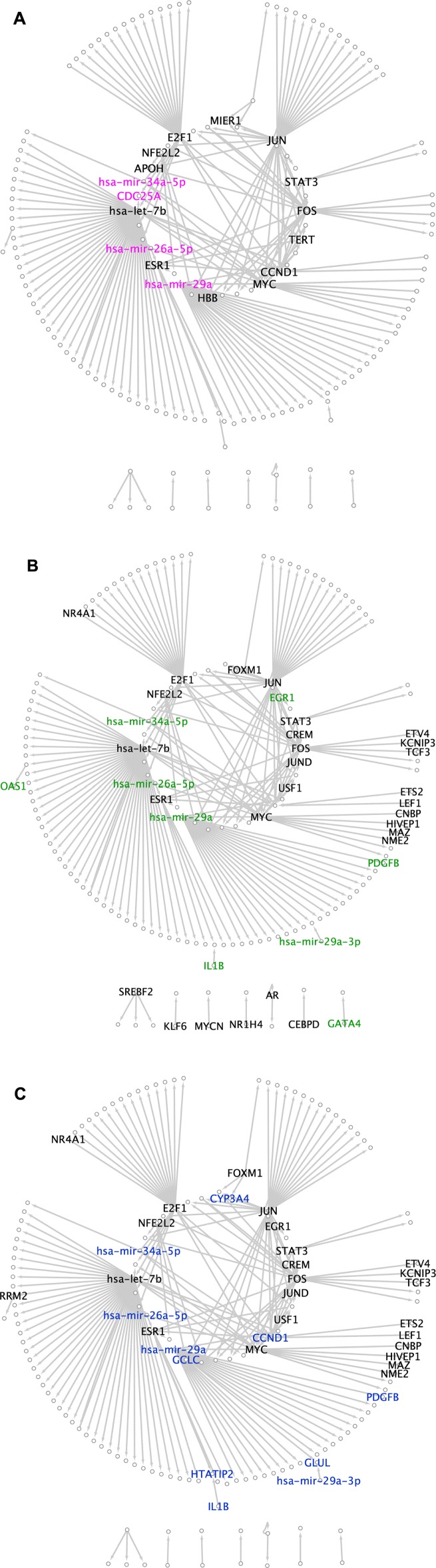
Topology consistency in the disease-specific networks for the LIHC dataset. **a** Top hub-degree genes and miRNAs colored pink. **b** MDS nodes colored green. **c** MCDS nodes colored blue. The black ones are the consistent genes and miRNAs between DESeq, edgeR, voom and VST including 13 out of 17 hubs, 28 out of 37 MDS and 24 out of 35 MCDS nodes selected by DESeq method

In the case of the LIHC dataset, analyzed by the DESeq method, 163 nodes and 199 edges make up the hepatocellular carcinoma disease-specific network. The hubs, MDS and MCDS of the network are visualized in Fig. 2.

In the case of the breast neoplasms, the network derived from BRCA dataset and the same method consists of 227 nodes and 302 edges. The TFmiR webserver also was used to construct disease-specific networks for the set of DE genes derived from edgeR, voom and VST input data. Additional file 1: Tables S3 and S7 show the number of nodes, edges, hubs, MDS and MCDS for the LIHC and BRCA datasets for the four methods, respectively.

### Topology consistency

We performed pairwise comparisons between the topological features of these networks, see Tables 1 and 2. The results demonstrate the percentage overlap of hubs, MDS and MCDS between the aforementioned analysis methods. As shown in the tables, DESeq has a comparable overlap with edgeR, voom and VST in both the studies, whereas the topological features of edgeR overlapped better with voom than VST.

**Table 1:** Pairwise comparison (percentage overlap) of hubs, MDS and MCDS for the LIHC dataset

Methods	edgeR	voom	VST
DESeq	82,84,77	88,81,74	82,81,71
edgeR	-	80,82,78	70,75,68
voom	-	-	87,92,95

Pairwise comparison of hubs(left), MDS(middle) and MCDS(right numbers) for the networks constructed from the set of DE genes detected by DESeq, edgeR, voom and VST methods in the LIHC dataset

**Table 2:** Pairwise comparison (percentage overlap) of hubs, MDS and MCDS for the BRCA dataset

Methods	edgeR	voom	VST
DESeq	96,83,81	91,80,79	96,83,80
edgeR	-	86,83,83	70,72,75
voom	-	-	83,85,88

Pairwise comparison of hubs(left), MDS(middle) and MCDS(right numbers) for the networks constructed from the set of DE genes detected by DESeq, edgeR, voom and VST methods for the BRCA dataset

**Fig. 3:**
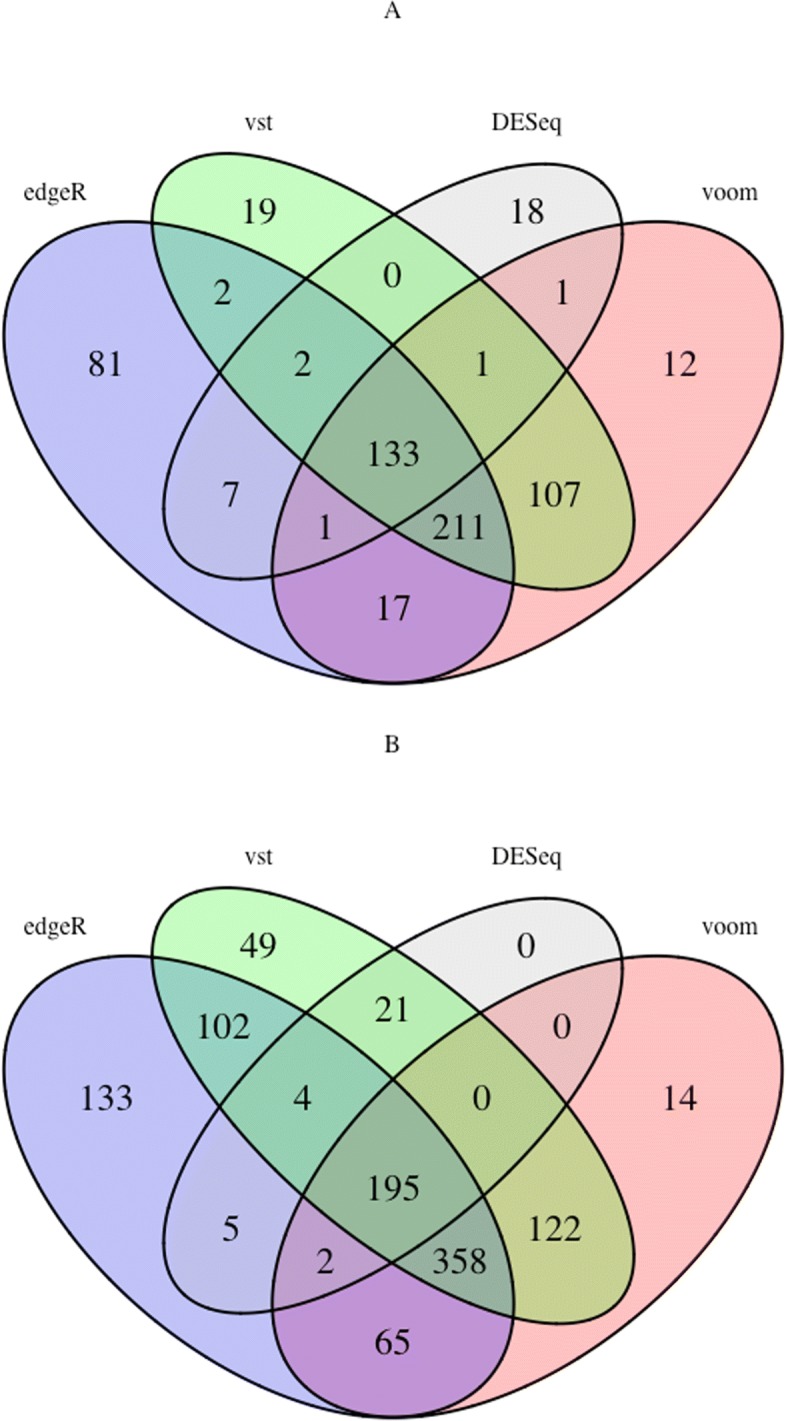
**a** Venn diagram for the number of overlapped network nodes between the results of DESeq and those from edgeR, voom and VST for the LIHC dataset. **b** same for the BRCA dataset

**Fig. 4:**
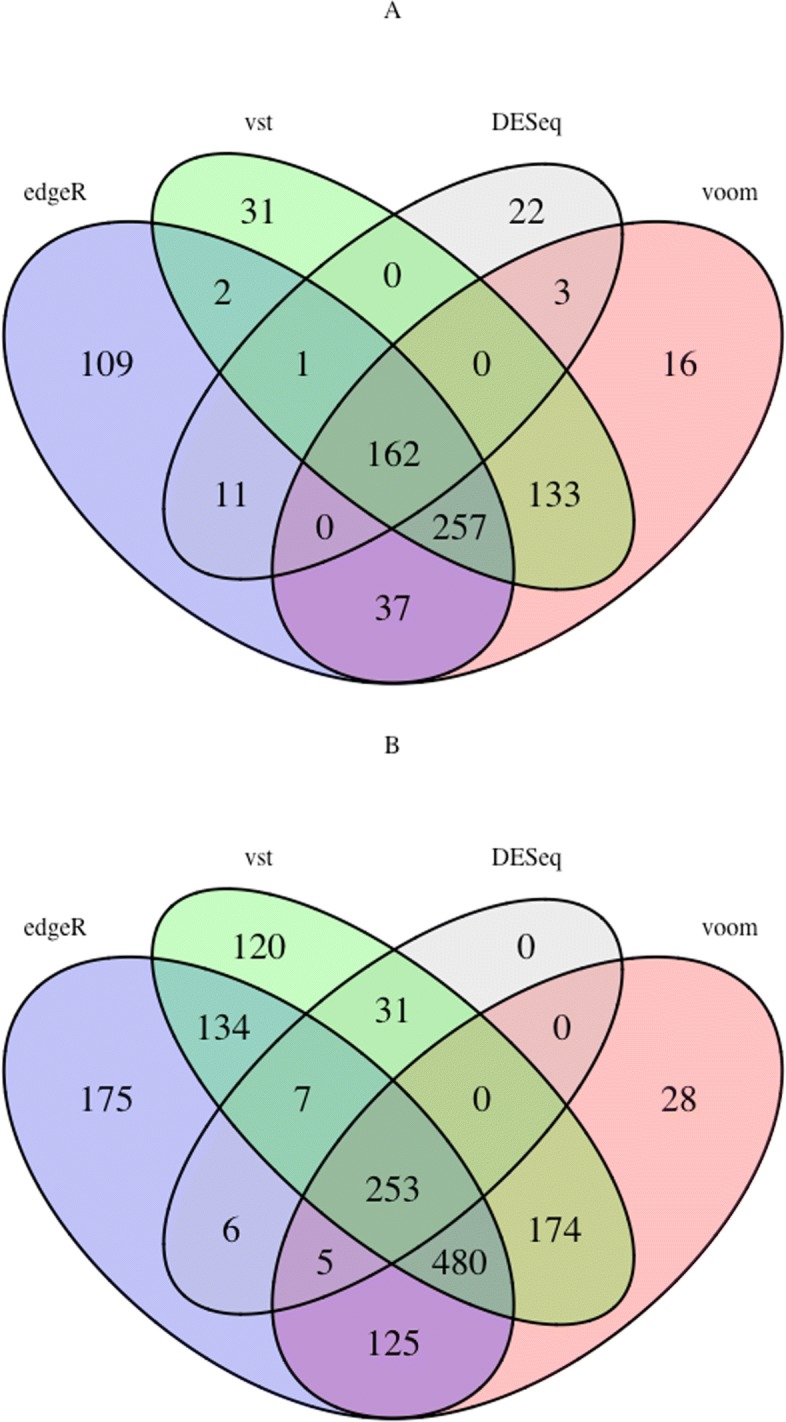
**a** Venn diagram for the number of overlapped network edges between the results of DESeq and those from edgeR, voom and VST for the LIHC dataset. **b** same for the BRCA dataset

Additional file 1: Tables S4, S5, S6 show the list of consistent genes and miRNAs that are common among all the methods for hepatocellular carcinoma and in Additional file 1: Tables S8, S9, S10 for breast neoplasms. The tables show a high number of consistent genes and miRNAs among the topological features of the methods. 13 out of 17 hubs selected by DESeq were identified by the other methods from the LIHC dataset and 20 out of 23 from the BRCA dataset. The common MDS and MCDS make up almost 70% to 75% of the selected MDS and MCDS by the DESeq method. The number of consistent topological features increases when we disregard the DESeq method, as it has the lowest number of DE genes, the smallest network size and subsequently the smallest set of hubs, MDS and MCDS among all the methods, see Additional file 1: Table S11. The absolute number of consistent topological features increases, but the fraction of consistent nodes remains near 70%. To investigate the consistency observation among topological features especially dominating sets, two Venn diagrams are visualized to describe the number of common network nodes and edges between the results of DESeq with edgeR, voom and VST for the LIHC and BRCA datasets, see Fig. 3 and Fig. 4. The figures illustrate a large number of overlapped network nodes and edges among their networks. 133 out of 163 and 195 out of 227 network nodes derived by DESeq method for LIHC and BRCA datasets were common among all the networks. Similarly, 162 out of 199 and 253 out of 302 edges were common among their network edges.

### Robustness of the results

**Fig. 5:**
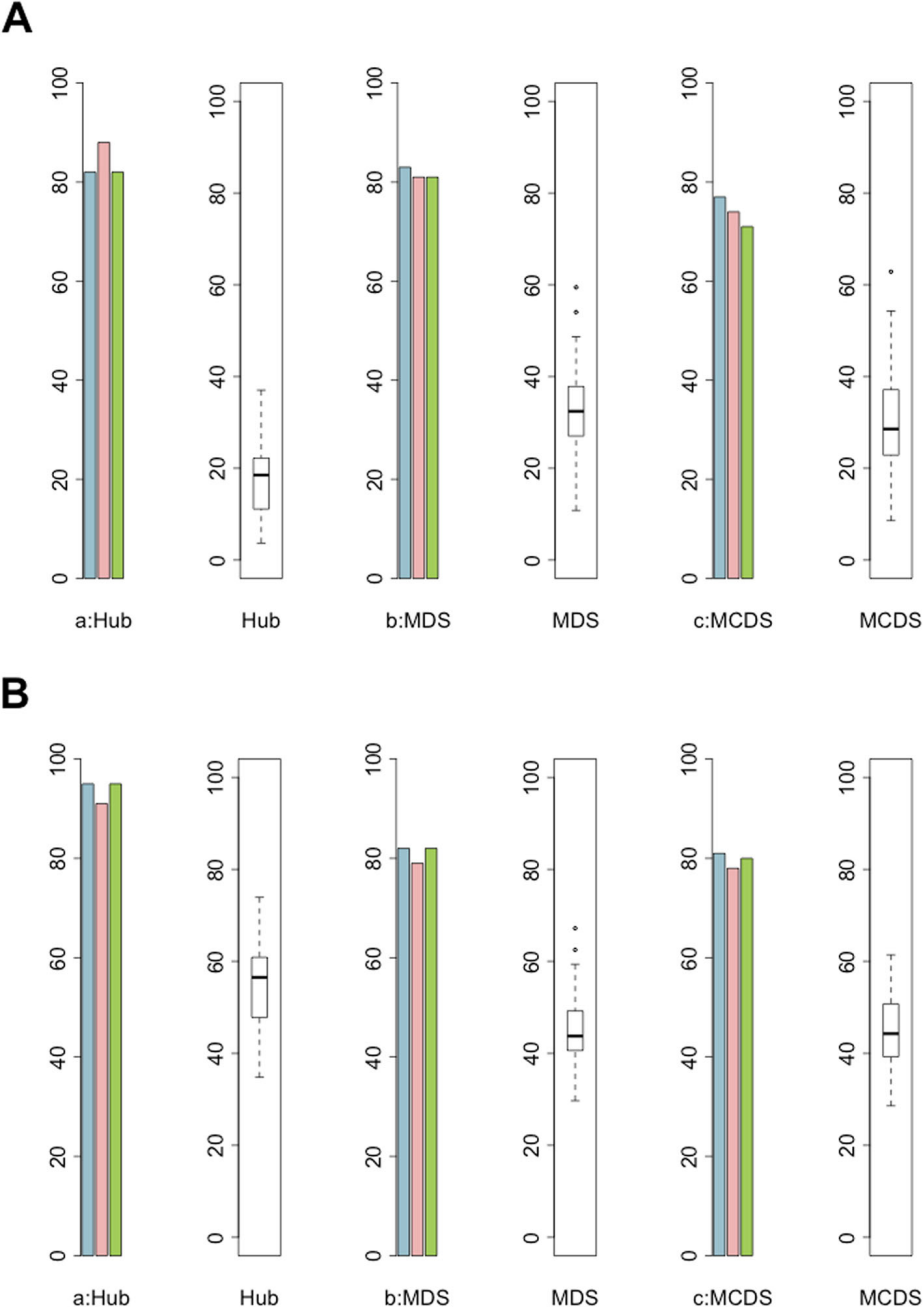
Robustness of the topological results. Barplots in panels (**a**) and (**b**) illustrate the percentage overlap of hubs, MDS and MCDS in the DESeq network with the other three (edgeR (blue), voom (red) and VST (green)) networks for the LIHC (**a**) and BRCA (**b**) datasets, respectively. Boxplots in panels (**a**) and (**b**) show the overlap of the three mentioned topological features of DESeq with 100 disease-specific networks derived of 11000 and 14000 randomly genes that were selected genes from the LIHC and BRCA datasets, respectively

To check the robustness and significance of the results, 100 random networks were constructed with 11000 and 14000 randomly selected genes as pseudo sets of DE genes, respectively. Related networks were constructed with TFmiR. Detection of hubs, MDS and MCDS were performed as explained before. The results of DESeq were compared with the other tools, edgeR, voom and VST. We used the widely used tool, DESeq [2] as the base line of comparison because it appears to be a very conservative method to detect the set of DE genes [4, 10]. Moreover, we realized from the previous experiments that DESeq contains the highest number of consistent topological features among all the methods. Barplots in Fig. 5a and b visualize the overlap percentage between DESeq and other methods, and boxplots in panels (A) and (B) show the percentage overlap of hubs, MDS and MCDS of DESeq with random networks for hepatocellular carcinoma and breast neoplasms, respectively. If one provides more than half of all human genes as input and generates a regulatory disease-specific network, one can expect that a considerable fraction of the real key genes is recovered by chance. In the two studied cases, between 20 and almost 60% overlap with the DESeq key genes. However, the results indicate that a random selection of nodes does not reach the same level of topological overlap compared to the topological overlap of DESeq with edgeR, voom and VST. Since none of the 100 random networks reached the values for the real networks, the significance is below *p*=0.01.

## Discussion

We have previously presented the webserver TFmiR [7] that uses lists of de-regulated mRNAs and/or miRNAs as input and complements these with experimentally validated and/or predicted regulatory interactions between TF-gene, TF-miRNA, miRNA-gene, and miRNA-miRNAs. These interactions are taken from well-known databases. The webserver thus generates differential co-regulatory subnetworks that may be further filtered to known disease conditions. One assumption made with TFmiR is that we assume that the regulatory links retrieved from the mentioned data sources are active in the particular cell types and conditions underlying the provided input sets of genes. Another assumption we make is that we consider the members of MDS and MCDS sets and hub genes as key players. In [8], we illustrated the plausibility of this concept on the examples of GRNs from *E.coli*, and *S.cerevisiae*, for mouse pluripotency and for human breast cancer. We have also presented algorithms to determine a MDS or a MCDS of key transcription factors and miRNAs that control all other (target) nodes of an input network [8] and we utilize these concepts to determine key regulators for a given network.

An important issue is whether different input sets of de-regulated mRNAs and/or miRNAs would lead to largely different results in downstream analyses. It is well-known as mentioned in [4] that there is little overlap between the results for de-regulated genes obtained by different tools that are all widely used. Since TFmiR is one such downstream analysis method, this would then also have important implications for our own work.

Here, we report an interesting and also re-assuring discovery, namely that key regulator sets consisting of hub genes/miRNAs as well as the members of MDS and MCDS are robust toward the set of input de-regulated genes/miRNAs. This is very good news for any scientist working on related types of downstream analyses.

Finally, we checked the biological relevance of the obtained results. For this, we considered the overlap of key genes obtained by downstream TFmiR analysis based on the input DE genes identified by the four analysis methods. Precisely, we considered the hub genes and microRNAs that are also members of the MDS and MCDS sets. For the LIHC dataset analysis, these are (LIHC.Hub.MDS.MCDS = hsa-let-7b, JUN, E2F1,FOS,MYC, ESR1, STAT3, NFE2L2). For the BRCA dataset, these are (BRCA.Hub.MDS.MCDS = JUN, hsa-mir-21, E2F1, TFAP2A, ESR1, STAT5A, ESR2, ETS2, hsa-mir-21-5p). All of these key genes and microRNAs have been described in the literature in the context of liver cancer [11–18] and breast cancer [19–27].

## Conclusion

In this work, we showed that disease-specific co-regulatory networks constructed with the TFmiR webserver from different sets of DE genes identified by different analysis methods contain largely overlapping sets of hubs, MDS and MCDS. Although the number of exclusive DE genes identified by each analysis method was considerable in liver cancer and in breast cancer, we found that topologies of the derived co-regulatory networks were highly consistent with respect to hub-degree nodes and MDS and MCDS (70-90%). This suggests that key genes identified in regulatory networks derived from DE genes are a robust basis for understanding diseases processes.

## Methods

Processed RNA-Seq data was obtained from [28] for matched tumor and normal samples of LIHC and BRCA datasets. We exploited the R packages of DESeq, edgeR, VST and voom methods [10, 29] to identify the respective sets of DE genes. edgeR and DESeq methods assume that the dispersion is a linear function of the mean based on a factor *α*, which is the same throughout the experiment and needs to be estimated from the data. edgeR estimates the common dispersion by considering the expression data of all genes of both groups across the samples. It calculates gene-wise dispersion using conditional maximum likelihood, conditioning on the total counts for that gene. Gene-wise dispersions are shrunk towards a common dispersion using an empirical Bayes procedure. Finally, the differential expression is assessed for each gene using an exact test similar to Fisher’s exact test, but adapted for data that have overdispersion [3, 30]. DESeq applies size factors to normalize the data (the median of the ratios of observed counts) to render samples comparable when the samples have been sequenced to different depths [2]. The *p*-value of a pair of observed count sums (*k*_*iA*_,*k*_*iB*_) is then the sum of all probabilities less or equal to *p*(*k*_*iA*_,*k*_*iB*_), given that the overall sum is *k*_*iS*_ [2]. The Variance Stabilizing Transformation (VST) takes the variance-mean dependence *w*(*q*) computed by DESeq and applies a transformation function to remove the dependency. The monotonous mapping function produces data whose variance is independent from the mean [2]. VST uses the limma package for performing the statistical tests and inferring the set of DE genes. voom (variance modelling at the observation level) attempts to estimate the mean-variance relationship robustly and without any parameter from data at the level of individual observations. It transforms count data to log-cpm (counts per million) values for the purpose of normalization [29]. To estimate the mean-variance trend at the level of individual observations, it computes a residual standard deviation for each gene. After fitting a robust trend to the residual standard deviations, the standard deviation for an individual observation is predicted by interpolating the standard deviation trend based on its predicted count size. Finally, the inverse square of the predicted standard deviation for each observation and log-cpm values are given to limma’s standard differentiation pipeline as input to obtain the set of statistically significant DE genes [29].

### Network construction with tFmiR

A TF-miRNA differential co-regulatory network was constructed using the TFmiR webserver for each set of DE genes [7]. TFmiR analyzes four different types of regulatory interactions, TF → gene, TF → miRNA, miRNA → miRNA, and miRNA → gene. As evidence for these interactions, TFmiR uses information from the following established repositories: TransFac, OregAnno, and MsigDB (for TF → gene links), TransmiR (for TF → miRNA links), mirTarBase, TarBase and miRecords (for miRNA → genes links), and PmmR (for miRNA → miRNA links). In the present case when only DE genes are provided as input, TFmiR identifies the set of missing miRNAs whose target genes as well as regulator TFs are significantly enriched within the input deregulated genes using the hypergeometric distribution function followed by the Benjamini–Hochberg adjustment with a cutoff value of 0.001 [7]. In this work, we focused on disease-specific networks and thus applied the filter for known disease-associated genes based on experimental evidence in TFmiR for hepatocellular carcinoma and breast neoplasms.

### Topology inference

For the constructed disease-specific networks involving TFs, microRNAs, and target genes, we selected the top 10% highest centrality nodes as hub-degree nodes. An MDS was calculated based on the ILP formulation described in [8], where a MDS in a regulatory network is the minimum number of regulatory genes and miRNAs that control the whole network. An MCDS was computed based on the heuristic approach mentioned in [8], where MCDS in a co-regulatory network is a set of genes and miRNAs that are connected and control the largest connected component (LCC) of the network.

## Supplementary information

**Additional file 1** The PDF file includes several figures and tables containing all the supporting materials for the manuscript.

## Data Availability

The raw data of two experiments were downloaded from the The Cancer Genome Atlas (https://cancergenome.nih.gov). The processed data and the programming scripts that we used to derive the set of DE genes are available at (https://github.com/maryamNazarieh/TopologyConsistency).

## Abbreviations

DE: Differentially expressed
NB: Negative binomial
LIHC: Liver hepatocellular carcinoma
BRCA: Breast invasive carcinoma
VST: Variance stabilizing transformation
MDS: Minimum dominating set
MCDS: Minimum connected dominating set
LCC: largest connected component
