# Supplementary material for: Topology Consistency of Disease-specific Differential Co-regulatory Networks
Source: BMC Bioinformatics. 2019 Nov 6;20:550. doi: 10.1186/s12859-019-3107-8 (PMC6833256; doi:10.1186/s12859-019-3107-8)
Supplement: Supplementary file 1 — Additional file 1 The PDF file includes several figures and tables containing all the supporting materials for the manuscript. [file 12859_2019_3107_MOESM1_ESM.pdf]

Table S1: Pairwise comparison (percentage overlap) of the sets of DE genes detected by DESeq, edgeR, voom and VST methods in the LIHC dataset. Shown in parentheses is the number of exclusive DE genes that were identified by either one of the methods not by both of them.

Methods	edgeR	voom	VST
DESeq	100% (7547)	94 (7219)	95 (6778)
edgeR	-	82 (4534)	82 (4739)
voom	-	-	96 (1135)

Table S2: Pairwise comparison (percentage overlap) of the sets of DE genes detected by DESeq, edgeR, voom and VST methods in the BRCA dataset. Shown in parentheses is the number of exclusive DE genes that were identified by either one of the methods not by both of them.

Methods	edgeR	voom	VST
DESeq	100% (9489)	97 (9723)	97 (8961)
edgeR	-	88 (3474)	89 (3924)
voom	-	-	97 (1594)

Table S3: Specifications of disease-specific networks for the LIHC dataset.

method	# nodes	# edges	# hubs	# MDS	# MCDS
DESeq	163	199	17	37	35
edgeR	454	579	46	87	98
voom	483	608	49	94	105
VST	475	586	48	93	99

Table S4: Consistent hub genes and miRNAs for the LIHC dataset.

hsa-let-7b, JUN, E2F1, FOS, MYC, CCND1, ESR1, TERT, STAT3, NFE2L2, HBB, APOH, MIER1

Table S5: Consistent MDS genes and miRNAs for the LIHC dataset.

NFE2L2, NME2, MAZ, MYCN, JUN, NR1H4, KCNIP3, NR4A1, TCF3, FOS, ETV4, ESR1, CREM, CNBP,
FOXM1, hsa-let-7b, STAT3, USF1, LEF1, SREBF2, HIVEP1, MYC, JUND, CEBPD, ETS2, KLF6, AR, E2F1

Table S6: Consistent MCDS genes and miRNAs for the LIHC dataset.

LEF1, MAZ, CREM, FOXM1, ETS2, MYC, HIVEP1, E2F1, hsa-let-7b, EGR1, JUN, RRM2, JUND, KCNIP3, CNBP, STAT3, NME2, FOS, ETV4, ESR1, USF1, NR4A1, TCF3, NFE2L2

Table S7: Specifications of disease-specific networks for the BRCA dataset.

method	# nodes	# edges	# hubs	# MDS	# MCDS
DESeq	227	302	23	64	70
edgeR	864	1185	87	145	173
voom	756	1065	76	144	169
VST	851	1199	86	147	168

Table S8: Consistent hub genes and miRNAs for the BRCA dataset.

JUN, hsa-mir-21, E2F1, TFAP2A, FOS, ESR1, CCND1, IFNB1, EGFR, STAT5A, IL6, ESR2, KIT, ERBB2, RARB, MYC, ETS2, hsa-mir-21-5p, STAT1, BRCA1

Table S9: Consistent MDS genes and miRNAs for the BRCA dataset.

EGR1, JUN, RARA, RARB, BMP6, hsa-mir-21-5p, ESR2, TCF7L2, TNFSF12, FOXA1, MEIS1, TCF3, PARP1, ETV5, ESR1, TFDP1, NR2F6, TRERF1, FOXM1, THRA, ZEB1, USF1, SRF, EFNA2, GBX2, LEF1, HEY2, E2F1, LMO2, hsa-mir-34a-5p, STAT5B, SREBF1, hsa-mir-21, WT1, TFF3, IRF7, TAL1, TEAD4, CEBPD, TFAP2A, ETS2, KLF6, hsa-mir-145-5p, NR3C1, JUND, NR4A1, STAT5A, RPA3

Table S10: Consistent MCDS genes and miRNAs for the BRCA dataset.

FOXA1, THRA, BRCA1, BRCA2, FOXM1, NR3C1, ETS2, CCND1, HEY2, TEAD4, SREBF1, hsa-mir-21-5p, LMO2, FOS, ETV5, TFDP1, TAL1, KIT, IRF7, TFF3, CEBPD, hsa-mir-145-5p, SRF, LEF1, EGFR, GBX2, CYP11A1, hsa-mir-21, E2F1, STAT5A, TFAP2A, TCF7L2, STAT5B, EGR1, JUN, JUND, IFNB1, CAV1, TNFSF12, hsa-mir-34a-5p, TRERF1, ESR1, BMP6, USF1, ESR2, VEGFA, NR4A1, IL6, EFNA2, WT1, RPA3, TCF3

Table S11: The number of consistent nodes among hubs, MDS and MCDS of edgeR, voom and vst.

dataset	# hubs	# MDS	# MCDS
LIHC	32	64	67
BRCA	55	101	122
